# Supplementary material for: Deletion of wfs1 Impairs Oligodendrocyte Precursor Cells Dorsal Distribution and Myelination Through the wfs1-hmgcs1 Axis in Zebrafish
Source: Biology (Basel). 2026 Aug 16;15(16):1402. doi: 10.3390/biology15161402 (PMC13510151; doi:10.3390/biology15161402)
Supplement: Supplementary file 1 [file biology-15-01402-s001.zip › File S1-Transcriptome sequencing data and differentially expressed genes (DEGs) in wfs1-- zebrafish at 5 dpf/2.KEGG/wfs1KOvsWT/DOWN/src/dre04810.html]

dre04810


- dre:445305

- Down regulated genes

445305\_1(-1.98867996431405)

- dre:767646

- Down regulated genes

767646\_1(-3.30449808454776)

- dre:566764

- Down regulated genes

566764(-2.60134676797662)
- dre:323285

- Down regulated genes

323285\_1(-3.43280624877224)

- dre:286739

- Down regulated genes

286739\_1(-2.60068066374091)

- dre:30429

- Down regulated genes

30429\_2(-1.78714384880949)

- dre:553493

- Down regulated genes

553493(-1.0476065195569)

- dre:793158

- Down regulated genes

793158(-6.94943122774273)

- dre:792699

- Down regulated genes

792699(-1.84389282152821)

- dre:436718

- Down regulated genes

436718\_1(-6.85571323128554)

- dre:100000252

- Down regulated genes

100000252\_1(-1.7718817168434)

- dre:100331581

- Down regulated genes

100331581\_1(-6.65159438165074)

- dre:557935

- Down regulated genes

557935(-1.87319484154862)
- dre:100536705

- Down regulated genes

100536705\_2(-6.17198352976631)
- dre:797491

- Down regulated genes

797491\_1(-1.09418588609297)

- dre:386856

- Down regulated genes

386856\_1(-1.99487147218035) novel.946(-6.7676180476835)
- dre:569566

- Down regulated genes

569566(-1.00953785382545)
- dre:571431

- Down regulated genes

571431(-6.42862549702529)

- dre:100005049

- Down regulated genes

100005049(-1.30748784788409)
- dre:560564

- Down regulated genes

560564\_2(-4.02056550705436)
- dre:103909601

- Down regulated genes

378480(-1.33145438810737)

- dre:57934

- Down regulated genes

57934(-1.25539807424297)

- dre:57934

- Down regulated genes

57934(-1.25539807424297)

- dre:57934

- Down regulated genes

57934(-1.25539807424297)

- dre:445305

- Down regulated genes

445305\_1(-1.98867996431405)

- dre:799354

- Down regulated genes

799354\_1(-1.21394100025012)

- dre:393611

- Down regulated genes

393611(-2.47215503276962)

Close
